# The relationship between felt stigma and non-fatal overdose among rural people who use drugs

**DOI:** 10.1186/s12954-024-00988-x

**Published:** 2024-04-06

**Authors:** Adams L. Sibley, Emma Klein, Hannah L.F. Cooper, Melvin D. Livingston, Robin Baker, Suzan M. Walters, Rachel E. Gicquelais, Stephanie A. Ruderman, Peter D. Friedmann, Wiley D. Jenkins, Vivian F. Go, William C. Miller, Ryan P. Westergaard, Heidi M Crane

**Affiliations:** 1 Department of Health Behavior, UNC Gillings School of Global Public Health, 170 Rosenau Hall CB #7400, 135 Dauer Dr, Chapel Hill, NC 27599 USA; 2 Department of Behavioral, Social, and Health Education Sciences, Rollins School of Public Health, Emory University, 1518 Clifton Road NE, Atlanta, GA 30322 USA; 3 OHSU-PSU School of Public Health, Oregon Health & Science University, 1810 SW 5th Ave, Suite 510, Portland, OR 97201 USA; 4 Division of Epidemiology, Department of Population Health, New York University School of Medicine, 180 Madison, New York, NY 10016 USA; 5 School of Nursing, University of Wisconsin–Madison, 4257 Signe Skott Cooper Hall, 701 Highland Avenue, Madison, WI 53705 USA; 6 Department of Medicine, University of Washington, Harborview Medical Center, 325 9th Ave, Box 359931, Seattle, WA USA; 7 University of Massachusetts Chan Medical School–Baystate and Baystate Health, 3601 Main St, Springfield, MA 01199 USA; 8 Southern Illinois University School of Medicine, 201 E Madison Street, Springfield, IL 62702 USA; 9 Department of Epidemiology, UNC Gillings School of Global Public Health, CB#8050, 3rd Floor Carolina Square, Chapel Hill, NC 27516 USA; 10 University of Wisconsin-Madison, 1685 Highland Avenue, 5th Floor, Madison, WI 53705-2281 USA; 11 Department of Medicine, University of Washington, Mail Stop 359931, Seattle, WA 98104 USA

**Keywords:** Opioids, People who use drugs, Overdose, Stigma, Self-stigma, Rural health

## Abstract

**Background:**

Drug overdose deaths in the United States exceeded 100,000 in 2021 and 2022. Substance use stigma is a major barrier to treatment and harm reduction utilization and is a priority target in ending the overdose epidemic. However, little is known about the relationship between stigma and overdose, especially in rural areas. We aimed to characterize the association between felt stigma and non-fatal overdose in a multi-state sample of rural-dwelling people who use drugs.

**Methods:**

Between January 2018 and March 2020, 2,608 people reporting past 30-day opioid use were recruited via modified chain-referral sampling in rural areas across 10 states. Participants completed a computer-assisted survey of substance use and substance-related attitudes, behaviors, and experiences. We used multivariable logistic regression with generalized estimating equations to test the association between felt stigma and recent non-fatal overdose.

**Results:**

6.6% of participants (*n* = 173) reported an overdose in the past 30 days. Recent non-fatal overdose was significantly associated with felt stigma after adjusting for demographic and substance use-related covariates (aOR: 1.47, 95% CI: 1.20–1.81). The association remained significant in sensitivity analyses on component fear of enacted stigma items (aOR: 1.48, 95% CI: 1.20–1.83) and an internalized stigma item (aOR: 1.51, 95% CI: 1.07–2.14).

**Conclusions:**

Felt stigma related to substance use is associated with higher risk of non-fatal overdose in rural-dwelling people who use drugs. Stigma reduction interventions and tailored services for those experiencing high stigma are underutilized approaches that may mitigate overdose risk.

## Introduction

The United States (US) overdose epidemic continues to pose a major threat to public health after three decades. 107,000 Americans died from drug overdose in 2021, the largest mortality rate on record and a 16% increase over 2020 [1], and more than 200,000 non-fatal overdoses were reported by emergency medical services in the past 12 months [2]. Rural areas in the US have felt the impact of the overdose epidemic since its beginning: non-medical prescription opioid use, the driver of the epidemic’s first wave, has historically been higher in rural areas, owing in part to greater unintentional injury and higher prescribing rates compared with urban areas [3–6]. As a result, rural counties outpaced urban counties in age-adjusted overdose death rates between 2007 and 2015 [7]. Though the rural-urban mortality gap has closed during the most recent waves of the epidemic, the state of the epidemic in rural America remains troubling: overdose deaths in rural areas increased five-fold (from 4.0 to 19.6 per 100,000) between 1999 and 2019 [7].

Reducing stigma has long been noted as a key priority in addressing and ending the overdose epidemic [8–11]. Stigma, a “distinguished and labeled difference” [12], leads to discrimination, social exclusion, and status loss among stigmatized people, including people who use drugs (PWUD) [13–15]. While public stigma toward mental illness is generally decreasing in the United States, shifts in attitudes toward PWUD are more mixed [16]. Notably, unlike most other health issues, substance use stigma is still legally sanctioned in the United States. While people with severe mental illnesses are afforded civil rights through the Americans with Disabilities Act and other federal statutes, these same protections are typically restricted for PWUD, e.g., limited to those in active treatment [8, 17]. As well, the use of most psychoactive substances remains criminalized, reinforcing associations between substance use and criminality, which explains, in part, why stigmatizing attitudes toward PWUD remain significant and seemingly intractable [18].

Stigma is a central component of the rural risk environment for substance-related harm [19–25]. PWUD have reported experiencing stigma from pharmacists while seeking syringe access, first responders and healthcare staff during overdose experiences, providers in substance use treatment settings, employers during job-seeking and employment, family and friends during social support seeking, and the community at large [26–34]. These experiences can lead to stigma becoming internalized and self-stereotypes being endorsed by PWUD, inducing the so-called ‘why try?’ effect, a feeling of futility about achieving one’s goals, including engagement in treatment [33, 35–39]. In rural areas and elsewhere in the US, substance use stigma is associated with multiple adverse outcomes, including depression, social isolation, familial rejection, suboptimal healthcare, and employment discrimination [9, 28, 33, 40–44].

Despite the multifarious psychosocial and health outcomes of substance-related stigma, the link between stigma and overdose in PWUD is understudied, with the exception of one urban study in Baltimore, Maryland demonstrating an association between stigma and recent self-reported overdose [45]. Building this evidence base is critical to determining the level of investment needed in anti-stigma programming as a possible tool for overdose prevention. Centrally, experiencing stigma is associated with risk factors for overdose, including increased temptation to use, severity of drug use, and injecting risk behaviors [46–48]. Stigma further reduces both the availability and utilization of substance use treatment and harm reduction services [22, 49–53], while major increases in uptake of and retention in these services are needed to meet current US government benchmarks for overdose reduction, with higher thresholds in rural than urban areas [54]. However, to our knowledge, no studies have explored the association between stigma and overdose among rural-dwelling PWUD. Given the salience of substance use stigma in rural areas, investigating this relationship is warranted [22, 23, 49, 55–60].

In this study we characterize the associations between felt stigma and self-reported non-fatal drug overdose in a cohort of PWUD living in rural counties across 10 states in the US. Felt stigma describes the cognitive outcomes of public stigma in PWUD that inhibit pursuing life goals, including help-seeking [61]. Felt stigma includes fear of encountering stigma from others and internalized stigma/shame [62]. Fear of enacted stigma describes the expected beliefs that others have about PWUD and the interpersonal consequences of these beliefs (e.g., discrimination). Internalized stigma is the acceptance and endorsement of public stigma towards one’s group and the behavior and cognitive consequences thereof [63–65]. We hypothesize that felt stigma will be associated with increased odds of non-fatal overdose in this population. A better understanding of this association will be useful to understanding the relative contribution of stigma to the epidemic and to better target overdose prevention efforts.

## Methods

### Study design

Participants (*n* = 3048) were recruited in 2018–2020 from study sites across 10 US states (Illinois, Kentucky, North Carolina, New England [Massachusetts, New Hampshire, Vermont], Ohio, Oregon, Wisconsin, West Virginia) as part of the Rural Opioid Initiative (ROI) Consortium [66]. Eligibility for enrollment was self-reported past 30-day use of any opioid “to get high” (e.g., heroin, prescription pain medications, etc.) or injection of any drug (the Wisconsin site required past 30-day injection of any drug). Modified chain-referral sampling, based closely on Respondent Driven Sampling (RDS), was used to recruit people into the study. Each study site identified between 42 and 279 “seeds” who met participant eligibility criteria and agreed to initiate recruitment chains by referring peers—other PWUD. In general, seeds were selected to represent the sex and racial/ethnic characteristics of the local population of eligible individuals. Seeds were given up to six coupons to distribute to peers. Each eligible and enrolled recruit was offered the opportunity to recruit 3–6 eligible peers who were part of their network, with the process continuing until the sample size goal was met. Incentives were offered for recruitment ($10-$20 per peer, depending on site) and for study participation ($40-$60). All studies included two or more counties in their study area, and chain-referral recruitment chains were initiated in each county.

Standardized surveys were administered using Audio Computer-Assisted Self-Interview, Computer-Assisted Self-Interview, or Computer-Assisted Personal Interview, depending on study site. Participants also completed rapid HIV, hepatitis C virus, and syphilis testing following the interview (standard testing was conducted at the West Virginia site). Participants received $25 for completing the survey and an additional $20 for the rapid tests. The protocol was approved by the IRB at each participating institution, and participants were covered by a federal Certificate of Confidentiality. A complete description of site-specific study procedures is provided in the ROI Consortium overview paper [66].

### Measures

#### Recent overdose

Participants were asked if they had ever experienced symptoms of an opioid overdose, described as “if you passed out, turned blue, or stopped breathing from using drugs.” If participants answered yes, they were then asked the date of their most recent overdose. Recent overdose was categorized as a dichotomous variable with a cut-point of 30 days.

#### Felt stigma

Felt stigma was assessed based on a 5-item scale adapted from Latkin et al. [41, 67]. Each item had a four-point response option ranging from “not at all” to “very much.” The questions, which elicited participants’ fear of enacted stigma (four items) and internalized stigma (one item), included: “How much do you fear you will lose your friends because you use drugs?”, “How much do you fear family will reject you because you use drugs?”, “How much do you think other people are uncomfortable being around you because you use drugs?”, “How much do you feel people avoid you because you use drugs?”, and “How much do you feel ashamed of using drugs?” To support construct validity of the scale, the items were submitted to exploratory factor analysis (EFA) using the *psych* package in R 4.3.3. In factor enumeration, scree plot analysis (one factor above the plot elbow), parallel analysis (one factor with eigenvalue greater than simulated random chance values), and the Kaiser-Guttman criterion (one eigenvalue > 1) each suggested a one-factor solution. EFA was next performed on one factor using maximum likelihood estimation. All items had factor loadings between 0.63 and 0.82, and the single factor accounted for 57% of the variance in the data. Cronbach’s alpha for the scale in the sample was 0.84. Scale items were summed as a composite stigma score and standardized as Z-scores for ease of interpretation during analysis.

#### Demographics

Covariates were selected to represent standard demographic characteristics, including age, gender, education, homelessness, recent incarceration, full-time work, and selling drugs. Homelessness was measured as participant report of experiencing any homelessness in the previous 6 months. Recent incarceration was dichotomized as yes/no based on participants reporting spending 1 or more day in jail or prison in the previous 6 months. Both full-time work (defined for participants as 40 h/week) and selling drugs were assessed as participants reporting their main sources of income in the previous 6 months.

Drug-related risk factors included any recent injection drug use, daily injection drug use, drugs injected (heroin, methamphetamines, fentanyl, speedball, i.e., opioid and a stimulant), binge drinking, total number of lifetime overdoses, positive screen for opioid dependence, and ever-possession of naloxone. Recent injection drug use was derived from a question asking participants the most recent date they injected drugs and dichotomizing yes for any injecting in the previous 6 months. Daily injection drug use was derived from a question asking participants how often they injected any drugs in the previous 30 days and dichotomizing yes for daily (or more frequent) injection. Injection of a specific drug was dichotomized as yes if participants reported injecting that drug at least one day in the previous 30 days. Binge drinking was dichotomized as yes if participants reported at least one day in the previous 30 days on which they consumed 5 or more alcoholic drinks (for males) or 4 or more alcoholic drinks (for females). Opioid dependence was measured using the 5-item Severity of Dependence Scale [68, 69].

### Analysis

Given the definition of overdose in the survey, we restricted the sample to participants who reported using opioids in the past 30 days (*n* = 2608, i.e., excluding participants only reporting injection use of other drugs). Baseline descriptive characteristics were calculated for those with recent overdose and those without recent overdose, and bivariate group differences were compared using Pearson’s chi-squared test for categorical variables and t-tests or Wilcoxon rank sum tests for continuous variables.

We accounted for missingness through multiple imputation using fully conditional specification under a missing at random assumption. All covariates were used in the imputation model, and 20 imputed datasets were created. Missingness by variable ranged from 0% (age) to 10.0% (speedball injecting). Multiple imputation reduces bias in parameter estimates compared with listwise deletion of cases with missing values [70].

Both unadjusted and multivariable logistic regressions were used to test the association between stigma and recent overdose. All covariates were retained in the multivariable models as they were identified *a priori* as theoretically important. In addition to the theory-based covariates, study site was retained as a fixed effect in the multivariable model. As a sensitivity analysis, and to support the construct validity of the composite stigma measure, we ran two additional multivariable models—one with only the four fear of enacted stigma items (summed and excluding the internalized stigma item), and one with only the internalized stigma item (dichotomized at “very much” given left skew in the response distribution). To aid inference about the temporal association between stigma and overdose (given that recent overdose may, in fact, precede felt stigma), we also conducted a sensitivity analysis using past-year overdose, rather than past-30-day overdose, as the outcome. It is expected that an attenuated association in this sensitivity analysis would lend some support to felt stigma preceding overdose. All models were estimated as generalized estimating equations clustering the standard errors at the RDS seed level to account for autocorrelation that may be present due to nesting within seeds [71]. Analyses were conducted using SAS 9.4 software (SAS Institute Inc., Cary, NC, USA).

## Results

### Sample characteristics

The analytical sample consisted of 2,608 rural-dwelling people who had used opioids within the past 30 days. Of these participants, 51.8% (*n* = 1352) reported having ever overdosed and 6.6% (*n* = 173) reported experiencing an overdose in the past 30 days. The average number of lifetime overdoses in the sample was 2.9. The mean age was 36 years and 57.3% of participants were male. Half (53.4%) of the sample reported experiencing homelessness at some point in the last 6 months and 41.5% had spent time in jail or prison in the last 6 months. Most participants (86.2%) had injected drugs in the past 6 months, and slightly more than half of participants (58.4%) reported injecting at least once per day. The most commonly injected drugs within the past 30 days were heroin (65.9%) and methamphetamine (59.7%). Benzodiazepine use was also common (52.5%), as was binge drinking (54.9%). The mean felt stigma score was 9.76 (range: 0–15, SD: 4.09). Approximately two-thirds (66.2%) of participants reported experiencing at least some level of all four fear of enacted stigma items, while 89.6% reported at least some internalized stigma. When evaluating stigma items dichotomized as “very much” vs. other, family rejection (52.6%) and shame (51.6%) were the most commonly reported.

### Bivariate analyses

In bivariate analysis, participants who reported an overdose in the previous 30 days had higher felt stigma scores than those without recent overdose (*p* < .001, Table 1). Participants with recent overdose were more likely to have experienced recent homelessness (*p* < .001) and incarceration (*p* < .001), more likely to sell drugs for income (*p* < .001), and more likely to have engaged in all measured substance use behaviors except binge drinking (*p* = .26) when compared with those without recent overdose. Higher proportions of those reporting recent overdose also reported ever carrying naloxone (*p* < .001) and screened positive for opioid dependence (*p* < .001).

**Table 1:** Baseline characteristics of 2608 people who use drugs from rural regions, stratified by overdose status

Variable	Opioid overdose in past 30 days (*n* = 173) n (%)	No opioid overdose in past 30 days (*n* = 2435) n (%)	Chi-Square p-value
Felt stigma Z-score, mean (SD)	0.43 (0.85)	-0.03 (1.00)	< .001^1^
Number of lifetime overdoses, median (IQR)	3 (5, 9)	0 (0, 2)	< .001^2^
Age			0.20
18–25	26 (15.0)	351 (14.4)	
26–35	79 (45.7)	961 (39.5)	
36–45	45 (26.0)	655 (26.9)	
Greater than 45	23 (13.3)	468 (19.2)	
Gender			0.44
Male	104 (60.1)	1383 (57.1)	
Female	69 (39.9)	1038 (42.9)	
Race/Ethnicity			0.61
White	144 (83.2)	1998 (83.0)	
Black	4 (2.3)	77 (3.2)	
American Indian/Alaskan Native	16 (9.3)	162 (6.7)	
Other races	4 (2.3)	72 (3.0)	
Hispanic/Latinx of any race	5 (2.9)	98 (4.1)	
Education			0.13
Less than high school	36 (20.8)	562 (23.1)	
High school	74 (42.8)	1161 (47.7)	
Greater than high school	63 (36.4)	710 (29.2)	
Homeless in past 6 months	123 (71.5)	1251 (52.1)	< 0.001
Main Source of income fulltime work	26 (15.2)	438 (181)	0.34
Main Source of income selling drugs	66 (38.6)	528 (21.9)	< 0.001
Incarcerated in past 6 months	91 (53.2)	961 (40.7)	< 0.001
Ever owned naloxone	127 (73.4)	136 (55.4)	< 0.001
Injection drug use in past 6 months	166 (96.0)	2077 (85.5)	< 0.001
Daily injection	134 (77.5)	1389 (57.0)	< 0.001
Injected heroin in past 30 days	158 (91.3)	1551 (64.1)	< 0.001
Injected fentanyl in past 30 days	102 (59.3)	752 (31.0)	< 0.001
Injected methamphetamines in past 30 days	131 (75.7)	1415 (58.6)	< 0.001
Injected speedball in past 30 days	119 (72.1)	908 (39.7)	< 0.001
Used prescription anxiety drugs in past 30 days	109 (63.4)	1253 (51.8)	0.003
Binge drinking in past 30 days	97 (59.2)	1230 (54.6)	0.26
Positive screen for opioid use disorder	157 (92.9)	1951 (82.7)	< 0.001

^1^ t-test for difference in means

^2^ Wilcoxon rank sum test

### Multivariable analyses

In unadjusted analysis, the composite felt stigma measure was significantly associated with odds of recent overdose (OR: 1.72, 95% CI: 1.43–2.07). This association was attenuated but remained significant when adjusting for all demographic and drug-related covariates: A one standard deviation increase in felt stigma was associated with a 1.47-fold increase in the odds of recent overdose (95% CI: 1.20–1.81, Table 2). Sensitivity analyses supported this finding. Examining the association between stigma and overdose using only the four fear of enacted stigma items (i.e., without the internalized stigma item), the association was nearly unchanged (aOR: 1.48, 95% CI: 1.20–1.83, *p* = .0002). Likewise, the association between internalized stigma and recent overdose was significant (*p* = .02), with those endorsing “very much” on the shame question having 1.51 times the odds of recent overdose compared with those reporting “somewhat,” “just a little,” or “not at all” (95% CI: 1.07–2.14). In the final sensitivity analysis, using past-year overdose as the outcome, the association between felt stigma and overdose was attenuated as expected (aOR: 1.11, 95% CI: 1.00-1.23, *p* = .053).

**Table 2:** Multivariable logistic regression analyses of recent overdose among 2608 people who use drugs from rural regions

Variable	aOR (95% CI)
Felt stigma (Z-score)	1.47 (1.20–1.81)***
***Sociodemographic covariates***	
Age	
18–25	Ref.
26–35	1.01 (0.62–1.66)
36–45	0.99 (0.57–1.71)
Greater than 45	0.86 (0.45–1.65)
Gender	
Female	Ref.
Male	1.30 (0.91–1.84)
Education	
Less than high school	Ref.
High school or GED	1.02 (0.65–1.60)
Some college or greater	1.72 (1.07–2.76)*
Homelessness^1^	1.57 (1.07–2.29)*
Full-time work^1^	0.87 (0.54–1.41)
Main income from drugs^1^	1.62 (1.12–2.35)**
Incarceration^1^	1.08 (0.76–1.55)
Race/ethnicity	
Non-Hispanic White	Ref.
Non-Hispanic Black	0.81 (0.27–2.43)
Non-Hispanic American Indian/Alaskan Native	1.24 (0.67–2.30)
Other races	0.78 (0.27–2.25)
Hispanic/Latinx of any race	0.68 (0.26–1.80)
***Substance use-related covariates***	
Number of lifetime overdoses	1.03 (1.02–1.05)****
Carry Naloxone	1.15 (0.76–1.72)
Injecting drug use^1^	0.77 (0.29–2.10)
Daily injection^2^	1.17 (0.75–1.82)
Injected drugs^2^	
Heroin	2.60 (1.23–5.47)*
Fentanyl	1.21 (0.81–1.82)
Methamphetamines	0.99 (0.57–1.72)
Speedball	1.95 (1.19–3.20)**
Benzodiazepine use^2^	1.12 (0.78–1.60)
Binge drinking^2^	1.23 (0.85–1.80)
Positive OUD screen	1.11 (0.55–2.23)
***Study site***	
Illinois	Ref.
Kentucky	0.73 (0.24–2.19)
North Carolina	1.16 (0.42–3.21)
New England	1.31 (0.50–3.43)
Ohio	2.41 (0.91–6.38)
Oregon	0.79 (0.22–2.85)
Wisconsin	0.93 (0.37–2.37)
West Virginia	0.53 (0.15–1.83)

^1^ Past 6 months

^2^ Past 30 days

*p < .05. **p < .01. ***p < .001. ****p < .0001

## Discussion

Felt stigma, including its components (internalized stigma and fear of enacted stigma), was significantly associated with recent non-fatal opioid overdose in this cross-sectional, multi-state sample of PWUD. Those reporting higher levels of felt stigma were more likely to have experienced an overdose in the previous 30 days independent of demographic and substance use-related factors, including injecting frequency.

These findings are consistent with previous work in Baltimore, Maryland. Latkin et al. found a similar magnitude of association between stigma and self-reported overdose in a majority urban-dwelling, African-American sample, although with different measures of recency (overdose < 1 year ago: aOR: 1.7, 95% CI: 1.1–2.7; overdose > 1 year ago: aOR: 1.5, 95% CI: 1.2–1.9) [45]. As in this previous study, we note a relationship between fear of enacted stigma and overdose. In rural areas, which tend to have smaller social circles and fewer treatment providers, the lack of anonymity may exacerbate concerns about discrimination and discourage treatment-seeking for fear of being identified in the community as a PWUD [23, 55, 72, 73].

A key difference between the current study and Latkin et al. is that we found a significant association between overdose and internalized stigma (Latkin et al. reported aOR: 1.2, 95% CI: 0.8–1.8) [45]. One possible explanation for this discrepant finding lies in different social perceptions and norms surrounding substance use across US geographies [74, 75]. The cultural and religious landscapes in many rural areas often emphasize addiction as a moral failing and encourage self-reliance and self-sufficiency over help-seeking and social support [56, 75–80]. The relationship of internalized stigma to psychosocial health and treatment-seeking may thus be stronger in rural than urban areas. This association needs further exploration in other locations.

On the whole, our findings demonstrate that felt stigma may play a contributing role to the risk of overdose in rural areas. Although our study cannot determine causality, the mechanisms through which stigma might impact overdose are poorly understood and merit further study. We offer two possible frameworks that could be examined in future research: a *psychological distress* pathway and a *stigma avoidance* pathway.

In the *psychological distress* pathway, felt stigma may lead to deteriorated mental health among PWUD, which in turn leads to unsafe drug use practices. Substance-related stigma is associated with worsening mental health, including depression, anxiety, hopelessness, distress, reduced self-esteem, poor sleep, suicidal ideation, and feelings of isolation and guilt [41, 48, 81–84]. In turn, poor mental health, including depression, anxiety, and suicidality, is associated with increased risk for non-fatal overdose [85–89]. According to the ‘why try’ effect, prosocial, health-protective behaviors are deemed futile in the face of discrimination [36, 90]. PWUD with poor mental health may be less able to engage in coping and other adaptive self-maintenance strategies (e.g., harm reduction), have an increased drive to relieve depression through psychoactive means, and experience inhibited risk perceptions [86, 88, 89, 91, 92]. Notably, many PWUD who are overdose survivors may experience ‘passive suicidal intent’ before overdosing—not wanting to die, but not caring about the risks either [88]. Resulting risk behaviors, like higher-than-usual doses, polysubstance use, faster injecting, and lack of drug checking (e.g., for contaminants like fentanyl) may be mechanisms to overdose in PWUD with poor mental health [22, 86, 88, 89, 92, 93]. Though the *psychological distress* pathway has not been explored among PWUD, depression mediates the association between HIV-related stigma and risk behaviors among people living with HIV [94].

A *stigma avoidance* pathway was previously discussed, in part, by Latkin et al. [45]. PWUD with heightened perceived and internalized stigma may behave in ways so as to avoid experiencing stigma and its manifestations (e.g., prejudice and discrimination) [95, 96]. This process may involve concealing drug use from potential stigmatizers in private (friends, family) and public (law enforcement, passersby) spaces. For example, PWUD may use drugs while they are alone [97, 98], a risk factor or overdose fatality [99, 100]. More broadly, site of consumption is a key factor in the risk environment for PWUD in both rural and urban settings, and use in public and semi-public spaces is associated with increased risk for overdose [19, 22, 45, 101–105]. It has been suggested that PWUD who use drugs in open-air spaces (like public parks or parked cars) may rush their consumption in order to avoid detection by police or passersby [22, 103, 106]. Homelessness was one of the few variables significantly associated with overdose in adjusted analyses in our study; it follows that those without housing—who must use in public out of necessity—may be more likely to engage in such risky, stigma-avoidant practices [107]. In addition to public spaces, PWUD may seek refuge from stigma in semi-public spaces, such as “trap houses” (buildings for buying and using drugs), where they are likely to encounter only other PWUD [22, 103, 108]. In previous studies, rural-dwelling PWUD who use drugs in trap houses have described feeling rushed to inject, peer pressured to experiment with different substances or doses, and compelled to share syringes [22, 105, 108], behaviors that can precipitate overdose [103, 109–112].

If stigma increases overdose likelihood through risky consumption behaviors like rushed injecting, then there is an imperative to reach PWUD experiencing high stigma with tailored overdose prevention and harm reduction outreach and services. Two related but distinct goals in this context are preventing non-fatal overdoses from occurring in the first place and, as importantly, preventing overdoses that do occur from becoming fatal. Several harm reduction strategies have proven essential to promoting safer consumption, including freely available test strips (to check drugs for unexpected adulterants like fentanyl) and sterile syringes (which discourage receptive syringe sharing) [113, 114]. Meanwhile, overdose education and naloxone distribution (OEND) initiatives are essential tools in preventing fatal overdose [115]. Finally, safe consumption sites, also known as overdose prevention sites, offer a controlled and supervised environment for drug use as well as access to sterile equipment, education, and support services [116, 117]. These sites could serve as a safer alternative for PWUD accustomed to using in semi-public environments like trap houses.

However, solutions are needed for PWUD who are reluctant to attend public harm reduction programs for fear of experiencing stigma. In this regard, it may be beneficial to consider social network approaches to harm reduction. Social networks are important settings for the diffusion of health-related behaviors, both positive and negative [118]. Thus, while networks can increase substance use-related risks, e.g., through the spread of bloodborne infectious diseases, they may also be harnessed to promote health-protective behaviors [118, 119]. Social networks could serve as low-stigma conduits for promoting safer behaviors through peer education, norm shifting, and resource sharing, including clean syringes and fentanyl test strips. Peer support has been demonstrated as an essential feature for increasing trust, legitimacy, and reach of overdose prevention initiatives [120]. Champions—those with high PWUD network centrality—could play a key role in changing harm reduction behaviors outside of institutional settings [121]. Champion-centered peer education was used successfully to reduce HIV risk behaviors and seroconversion in networks of people who inject drugs in Ukraine [122]. Social networks may further provide a source of social support, which can reduce perceived and internalized stigma in PWUD and in turn improve mental health [81, 123, 124]. PWUD with high community attachment to their substance-using networks exhibited lower internalized stigma in one previous study [119]. Social support resources may be particularly valuable for PWUD with recent onset of substance use, as stigma beliefs are more tractable early on in the course of stigmatized conditions [125]. Given strong kinship and social networks in rural areas that often proliferate drug use risk behaviors, these durable ties may likewise be appropriate channels for promoting harm reduction and anti-stigma programming [75, 126].

Recognizing that not all PWUD will have robust networks, however, additional services are appropriate. Especially for those who prefer to use drugs alone—in the absence of bystanders who can administer naloxone or alert emergency services—one key consideration is preventing overdoses from becoming fatal. Recent phone- and app-based innovations, in which a PWUD connects anonymously to a person who can alert emergency services in case of an overdose, have demonstrated good acceptability among PWUD [127]. Third-party solutions (e.g., Never Use Alone, Brave) are available in the United States, while Canada has implemented a government-sanctioned overdose monitoring hotline [128].

It is crucial to acknowledge that the onus must not lie solely on PWUD to manage the consequences of stigma. Substance use is embedded in a social context of prohibition, criminalization, and legally sanctioned discrimination that dates back at least to the Harrison Narcotics Tax Act of 1914 and has been concretized through decades of regressive policies and a ‘war on drugs’ that punishes and socially devalues PWUD [129, 130]. Policy reform centered on decriminalization and civil protections for PWUD should be considered a priority in the primary prevention of substance use stigma.

This study has some limitations. Participants self-reported recency and frequency of non-fatal overdose. Recall bias may be a source of systematic measurement inaccuracies: It is possible that some participants engaged in telescoping, a tendency in survey response to report events more recently than they actually occurred [131], leading to an overcount of recent overdoses in the sample. However, it has been noted that recall bias diminishes proportionally with the salience of the event [132]. Overdoses are memorable events, producing trauma and grief responses [133, 134], and it is reasonable to assume that recall of recent such events is strong. Further, recent research suggests that standardized, administrative records like hospital diagnostic codes may severely undercount non-fatal overdose as compared with self-report [135].

As data collection was cross-sectional, temporality cannot be established. Rather than a unidirectional association, it is possible that overdose experiences and felt stigma are mutually constitutive. For instance, participants who sought medical care for recent overdose likely encountered law enforcement, emergency medical technicians, or healthcare providers, groups commonly reported to enact stigma against PWUD [27, 30, 32, 56, 136]. Experiences of prejudice or discrimination in these settings may have increased perceptions and internalization of stigma among some participants. It is possible that a feedback loop operates between these two constructs [137]: while stigma may increase the risk of overdose through the *distress* and *avoidance* pathways discussed above, stigmatizing experiences in the aftermath of an overdose may in turn magnify these very pathways, increasing risk for future overdose. One study of children impacted by HIV/AIDS in China demonstrated such a bidirectional, longitudinal relationship, with path analyses indicating that enacted stigma increased depressive symptoms, which in turn increased perceived stigma, and finally enacted stigma [138]. Our sensitivity analysis using a longer period of overdose recency demonstrated an attenuated association, lending some limited support to stigma preceding overdose in this cross-sectional study. Nonetheless, temporal evidence for causality is weak, and further longitudinal research is warranted to disentangle the causal relationship between stigma and factors precipitating overdose risk, as well as how PWUD stigma-related beliefs and management strategies change through time.

## Conclusions

This large, multi-site study demonstrates an association between felt stigma and recent overdose in PWUD living across several diverse, rural areas of the US. Non-fatal overdose has a dose-response relationship with subsequent fatal overdose; addressing known risk factors for non-fatal overdose is thus critical to preventing overdose deaths [139, 140]. Further work is needed to understand the psychosocial mechanisms that might underlie the association between stigma and overdose in order to interrupt this pathway. Given growing concerns with polydrug overdose, future studies should also explore this association among PWUD using non-opioid drugs with potential for contamination [141, 142]. Nonetheless, stigma reduction interventions—as well as tailored services for PWUD experiencing high felt stigma—are likely underutilized approaches that may decrease the risk of overdose.

## Data Availability

The data that support the findings of this study are not openly available due to the sensitive nature of the data but are available from the corresponding author upon reasonable request.

## Abbreviations

OEND: Overdose Education and Naloxone Distribution
PWUD: Person/People Who Uses Drugs
RDS: Respondent Driven Sampling
ROI: Rural Opioid Initiative
US: United States
